# Effects and metabolism of fish collagen sponge in repairing acute wounds of rat skin

**DOI:** 10.3389/fbioe.2023.1087139

**Published:** 2023-02-22

**Authors:** Lei Wang, Yan Qu, Wenjun Li, Kai Wang, Song Qin

**Affiliations:** ^1^ The Affiliated Hospital of Weifang Medical University, Weifang, China; ^2^ Key Laboratory of Biology and Bioresource Utilization, Yantai Institute of Coastal Zone Research, Chinese Academy of Sciences, Yantai, China; ^4^ Department of Orthopedics, The Affiliated Yantai Yuhuangding Hospital of Qingdao University, Yantai, China; ^3^ University of Chinese Academy of Sciences, Yantai, China

**Keywords:** materials, collagen sponge, defect area, repair, fluorescent tracer

## Abstract

**Objective:** Study the repair effect of tilapia collagen on acute wounds, and the effect on the expression level of related genes and its metabolic direction in the repair process.

**Materials and methods:** After the full-thickness skin defect model was constructed in standard deviation rats, the wound healing effect was observed and evaluated by means of characterization, histology, and immunohistochemistry. RT-PCR, fluorescence tracer, frozen section and other techniques were used to observe the effect of fish collagen on the expression of related genes and its metabolic direction in the process of wound repair.

**Results:** After implantation, there was no immune rejection reaction, fish collagen fused with new collagen fibers in the early stage of wound repair, and was gradually degraded and replaced by new collagen in the later stage. It has excellent performance in inducing vascular growth, promoting collagen deposition and maturation, and re-epithelialization. The results of fluorescent tracer showed that fish collagen was decomposed, and the decomposition products were involved in the wound repair process and remained at the wound site as a part of the new tissue. RT-PCR results showed that, without affecting collagen deposition, the expression level of collagen-related genes was down-regulated due to the implantation of fish collagen.

**Conclusion:** Fish collagen has good biocompatibility and wound repair ability. It is decomposed and utilized in the process of wound repair to form new tissues.

## 1 Introduction

Skin tissue defect caused by various reasons is a common clinical problem. The key to treatment lies in the rapid reconstruction and functional recovery. Wound repair mainly includes coagulation period involving blood clot formation, vasoconstriction, initiation of endogenous and exogenous coagulation processes; inflammatory period involving resistance to foreign microbial invasion, tissue debris removal, fibroblast proliferation and differentiation, collagen synthesis; repair period involving epithelial regeneration and granulation tissue formation, etc (Zhao and Tang, 2017). In this process, coagulation, inflammatory response, angiogenesis, granulation tissue formation and tissue remodeling are involved, it is a complex process involving multiple factors and regulated by multiple mechanisms. Problems in any link will lead to non-healing of wounds, infection, damage to blood vessels and nerves, and impaired autoimmune functions (Lin et al., 2012; De Almeida et al., 2013; Yamamoto et al., 2014). At the wound site, macrophages mainly clean the damaged cells and tissue debris. Fibroblasts are responsible for initiating angiogenesis, epithelialization, and collagen production; Epithelial cells mainly promote wound re-epithelialization. TGF-β, TNF-α, EGF, PDGF, VEGF, FGF, IL-1, IL-6, IL-8, IL-12, and other factors are involved in the process of inflammation and repair, guiding various types of cells to enter the wound site, and regulating epithelialization, collagen accumulation and angiogenesis. How to interfere with the process of wound healing and improve the speed of wound healing and repair effect has become a research hotspot. The traditional methods of treating skin defects are mainly debridement, disinfection, gauze bandaging, etc. The treatment cycle is long, the curative effect is poor, and it cannot meet the repair and treatment of different types of wounds. At the same time, in the process of traditional treatment, due to the need to change materials and other operations, not only bring pain to patients, but also increase the workload of medical staff. Once it develops into a chronic or infectious wound, the cost of treatment will be greatly increased (Castillo-Briceno et al., 2011; Banerjee et al., 2014; Holmer et al., 2014). According to clinical investigation, for patients with diabetic foot, each treatment cycle is about 1–2 months, and the cost is between $2,000 and $20,000. Therefore, how to select appropriate wound repair materials for rapid and effective wound repair is the common demand of clinical workers and patients. Among them, with the continuous development of tissue engineering, wound repair materials that can replace defective skin, provide short-term barrier protection, create a suitable healing environment, and effectively prevent infection have been widely studied (Caballé-Serrano et al., 2020; Salvatore et al., 2020; Schönborn et al., 2020; Yu and Wei, 2021).

Collagen which is the main component of extracellular matrix has good biocompatibility, low immunogenicity, biosafety, mechanical properties, etc. (Hernández Rangel and Martin Martinez, 2021). It is the raw material of various biomedical materials such as wound repair sponge, repair gel and repair film. Because of its excellent ability to promote cell crawling and growth, and good repairing effect, it is widely accepted in clinic (Miri et al., 2016; Wang et al., 2020; Cruz et al., 2021; Li et al., 2021; Yeung and Kelly, 2021). At present, the collagen used in clinic is mainly derived from terrestrial animals, but the application of terrestrial animal collagen is limited due to the risk of carrying zoonotic diseases such as foot-and-mouth disease and bovine spongiform encephalopathy (BSE) (Lim et al., 2019). Collagen derived from marine organisms has only minor differences in amino acid composition compared with terrestrial animals, and is highly homologous to collagen from bovine and porcine (Yamamoto et al., 2014; Chen et al., 2019). Collagen from fish has a typical (Gly-X-Y) _n_ repeat structure, containing high proportions of glycine, hydroxyproline, and low proportions of cysteine, tyrosine, histidine, hydroxylysine and methionine, etc., with low antigenicity and strong hydrophilicity (Jafari et al., 2020). Studies had shown that collagen derived from marine organisms has good cell adhesion ability, could induce the secretion of various factors, promote re-epithelialization and dermal reconstruction, and effectively accelerate wound healing. Therefore, it can be used as a new source of collagen for the preparation of skin wound repair materials for the treatment of severe wounds (Copes et al., 2019; Lin et al., 2019; Meyer, 2019; Wu et al., 2020).

Preliminary studies had found that dressings made of type I collagen extracted from tilapia have a good effect on promoting repair in the treatment of rat skin damage caused by burns. In order to observe and research the collagen sponge-induced cell crawling and proliferation ability, wound repair effect, changes in the wound, and metabolic conditions, the repair effect of the rat wound model was observed in this study. At the same time, the collagen was labeled with fluorescein to observe the change and fate during the repair process. Provide theoretical basis and data support for clinical application.

## 2 Materials and methods

### 2.1 Materials

Fish collagen sponge (which was extracted by acid-enzyme combination from tilapia skin (Pal et al., 2016)), Bovine collagen sponge (Wuxi Beidi Bioengineering Co., LTD.), SD rat (Jinan Peng Yue Animal Breeding Center), Immunohistochemical kit (Beijing Solaibao Technology Co., LTD.), FGF antibody, VEGF antibody, Col I antibody (Abcam), Col III antibody (Amyjet), FITC (Thermo), CCK-8 kit (Beijing Solaibo Technology Co., LTD.), Live cell/dead cell double staining kit (Calcein-AM/PI), L929 (mouse fibroblast, Qingqi (Shanghai) Biotechnology Development Co., LTD.), Roswell Park Memorial Institute (RPMI) 1640 (Thermo), Glutaraldehyde (Maclin), Hematoxylin-Eosin (HE) Stain Kit (Beijing Solaibao Technology Co., LTD.), Masson’s Trichrome Stain Kit (Beijing Solaibao Technology Co., LTD.).

### 2.2 Methods

#### 2.2.1 Characteristics and structure observation of fish collagen sponge

The collagen of tilapia was extracted by the method of acidase combination, and after moderate freeze-drying, it was prepared into collagen concentrate, placed in a mold, and then freeze-dried to obtain collagen sponge with certain spatial structure. The collagen sponge was prepared into a 0.3✕0.5✕0.5 mm^3^ cube, sprayed with gold (7Pa, 15mA, 200s), and its spatial structure, porosity, pore size, etc. were observed under a scanning electron microscope to determine whether its pore structure was suitable for cell adhesion and crawling.

#### 2.2.2 Cell culture

Weight 5 g sterilized fish collagen sponge, put it into 500 ml sterile triangular bottle in ultra-clean workbench, add 300 ml RPMI-1640 culture medium, placed the triangular bottle into constant temperature air shaking Table 1, set the temperature at 37°C, rotate speed at 50 RPM, and extracted for 12 h to obtain material extract. L-929 mouse fibroblasts were cultured with RPMI-1640 system containing 10% FBS, and then cultured for 48–72 h to reach the exponential growth period. After that, the L-929 mouse fibroblasts were inoculated into a new culture flask at a concentration of 4✕10^4^/ml. After 24 h, the original culture medium in the culture flask was removed, and 15 ml of RPMI-1640 culture medium was added into the control flask, and 15 ml of culture medium containing 100% extract, 50% extract and 25% extract was added into the experimental group. After 24 h, 3 culture bottles were taken from each group for morphological observation and counting. After statistics, the relative proliferation rate of cells was calculated. The cytotoxicity of collagen sponge was determined by referring to the cytotoxicity evaluation grading standards (Table 2). After L-929 mouse fibroblasts in logarithmic growth phase were obtained using the same method, the sponges and cells were put into a 48-well plate at the same time, and on the 1st, 3rd and 7th day, cells were stained to observe the live/dead state and growth density of cells in the material. After the material co-cultured with cells was freeze-dried, the adhesion, crawling and growth of cells on the surface and inside of the material were observed by scanning electron microscope.

**TABLE 1:** Primer sequence.

Primer	Sequence (5′-3′)	Length
Col I-F	AAA​GAT​GGA​CTC​AAC​GGT​CTC	150bp
Col I-R	CAG​GAA​GCT​GAA​GTC​ATA​ACC​A	
Col III-F	GCC​TCC​CAG​AAC​ATT​ACA​TAC​C	192bp
Col III-R	TGT​CTT​GCT​CCA​TTC​ACC​AG	
Actin-F	TCC​TCC​TGA​GCG​CAA​GTA​CTC​T	153bp
Actin-R	GCT​CAG​TAA​CAG​TCC​GCC​TAG​AA	

**TABLE 2:** RT-PCR reaction system.

Component	Usage amount
primer F	0.5μl
primer R	0.5μl
2x Taq PCR Master Mix	10μl
2x Taq PCR Master Mix	7μl
RNase-free H_2_O	1μl
cDNA	20μl
**total volume**	0.5μl

#### 2.2.3 Preparation of rat skin defect model

90 SD rats were randomly divided into three groups: A, B, C, each 30. The rats were anesthetized with 2% pentobarbital sodium at a dose of 0.2 ml/100 g. The 3*3 cm^2^ area on the back of each rat was depilated. After the treatment, a trephine with a diameter of 1.5 cm was used to take the full-thickness skin from the exposed skin, to create a rat skin defect model. The wound was cleaned with normal saline, and after povidone-iodine disinfection, group A was filled with fish collagen sponge (FCS); group B was filled with bovine collagen sponge (BCS), group C was disinfected with disinfectant every 2 days, and the wound was covered with conventional medical gauze, a medical bandage was used to wrap the rat’s body 2 times to secure the gauze. Each rat was injected intramuscularly with 400,000 units of penicillin in the thigh muscle. All rats were fed under normal conditions and drank normally after operation.

#### 2.2.4 General observation

After the operation, the inflammatory reaction, granulation growth, wound growth, and surrounding redness and swelling of wound sites were observed and recorded every day; at the same time, the degradation, and utilization of materials implanted in the wound sites of each group were observed. The individual appearance time of the complete wound filling, the individual appearance time of complete wound healing, and the complete recovery time of all individuals were recorded in each group of rats, and the comparison and analysis were carried out among each group.

#### 2.2.5 Electron microscope observation

After 24 h and 48 h, the rats were anesthetized with 2% pentobarbital sodium at a dose of 0.2 ml/100 g. Tissue samples were taken from the defect area, and washed with PBS (PH7.0). After that, they were fixed with 2.5% glutaraldehyde solution for 8 h, freeze-dried at low temperature, sprayed with gold, and the dynamic changes of collagen fibers, elastic fibers and other scaffold structures in each sample were observed by scanning electron microscope, as well as the ingrowth of blood vessels and wound remodeling.

#### 2.2.6 Histological observation

After modeling, the rats in each group were mercy killed by pentobarbital sodium injection at a dose of 150 mg/kg on the 1st, 3rd, 7th, and 14th day. After measured the size of the wound area, the entire wound defect area tissue was cut along 0.3 cm outside the edge of the wound defect area and from the inside to the muscle layer. After fixation, routine paraffin fixation and sectioning, referred to the procedure of Hematoxylin-Eosin (HE) Stain Kit and Masson’s Trichrome Stain Kit, HE staining (dewaxing, cleaning, hematoxylin staining, acetic acid differentiation, reverting blue, eosin staining, dehydration, sealing) and Masson staining (dewaxing, cleaning, hematoxylin staining, cleaning, re-red by lichunhong acid liquid, glacial acetic acid leaching, phosphomolybdic acid differentiation, aniline blue staining, washing, dehydration, sealing) were performed; HE staining was used to observe the changes of cell types, cell morphology, tissue structure, blood vessels, nerves, etc. During the wound healing process. Mouse skin collagen, new collagen, changes in implant materials, etc. was observed through Masson staining.

#### 2.2.7 Immunohistochemical analysis

Immunohistochemical analysis of all pathological sections was performed according to the following steps: 1) Dewaxing: Paraffin sections of all samples were immersed in xylene, anhydrous ethanol, 95% ethanol, 85% ethanol, 75% ethanol and distilled water successively for dewaxing treatment, then treated with 3% hydrogen peroxide at room temperature for 5 min, and then cleaned with PBS for 3 times. 2) Antigen repair: Transfer the slices that have completed the dewaxing process into citrate buffer and heat them in a 92°C-water bath for 45 min to fully expose the antigens. 3) Reaction with primary antibody: 5% BSA sealer was added to the surface of sections to cover the tissues, and then placed in a moisturizing temperature chamber at 37°C for 30 min. After incubation, the tissue was dried. Col I, VEGF and FGF antibodies were added to different sections in each group respectively, and incubated in a moisturizing temperature chamber at 37°C for 2 h 4) Reaction with the second antibody: After the reaction of the first antibody was completed, the sections were taken out, all the anti-rat IgG labeled by HRP was added, and then the slices were placed in a moisture incubator for incubation at 37°C for 30 min. After cleaning with PBS, DAB chromogenic solution was used for color development. 5) Restaining: the sections were restained with hematoxylin, dehydrated, and then sealed with neutral gum after drying. The expression levels of three factors in different time points and different material samples were observed.

#### 2.2.8 Fluorescent labeling of fish collagen

Diluted the high-concentration fish collagen solution to 1–5 mg/ml with sodium bicarbonate buffer (pH 9.8), added into the dialysis bag, and placed the dialysis bag into sodium bicarbonate buffer containing 0.1 mg/ml FITC (pH 9.8), dialyzed with stirring for 12 h at 4°C in the dark. Then replaced the external buffer with PBS to stop the reaction, and changed PBS several times during the period until A480 nm was 0. Took 0.2 ml the labeled protein out, added 2.8 ml PBS, measure A490/A280, and calculated FITC/Protein (F/*p*-value), to identify the quality of the label.

#### 2.2.9 *In vivo* fluorescence observation

24 SD rats were randomly selected and divided into two groups and were anesthetized with 2% pentobarbital sodium at a dose of 0.2 ml/100 g. After the back was depilated, a full-thickness skin defect model with a diameter of 0.5 mm was made on their backs with a scalpel. One group was implanted with FITC-labeled fish collagen sponge, and the other group was implanted with FITC fluorescein. After the operation was completed, 3 rats were randomly selected from each group on the 1st, 3rd, 7th, and 14th day, observed the location of the wound, the changes of the fluorescence intensity around the wound, and whether there was fluorescence, the appearance site and fluorescence intensity through intravital fluorescence imaging system, under the conditions of excitation wavelength of 495 nm and emission wavelength of 519 nm, to judge the dynamic change process of fish collagen in and around the wound, and the dynamic metabolic process in animals.

#### 2.2.10 *In situ* fluorescence observation

After fluorescence detection *in vivo* of rats 14 days after surgery, the rats were mercy killed by pentobarbital sodium injection at a dose of 150 mg/kg and wound tissue was completely removed and cleaned with PBS for 3 times to prevent the observation results from being affected by fluorescein adhesion to surrounding tissues or surfaces. Put the material into a frozen slicer quickly, and then freeze the slicing with a thickness of 10 μm. The frozen section was placed under the confocal laser microscope under the condition of light protection, and fluorescence observation was conducted under the conditions of excitation wavelength of 495 nm and emission wavelength of 519 nm, to observe whether there was fluorescence, fluorescence intensity and fluorescence position in the internal structure of the tissue at the wound site. After observation, the corresponding frozen sections were taken for immunohistochemical reaction of Col I and Col III collagen. The immunohistochemical observation results were compared with fluorescence photos taken by laser confocal microscope for *in situ* analysis, and the tissue structure, collagen fiber state and collagen type at the fluorescence location were observed.

#### 2.2.11 Transcriptome analysis

Eighteen eight-week-old SD rats were randomly divided into two groups. The acute wound model was made using the same model making method as in 1.2.3. Sterile fish collagen sponge was implanted in the experimental group, while wound disinfection was only performed in the self-healing group. At 1, 3 and 7 days postoperatively, the rats were mercy killed by pentobarbital sodium injection at a dose of 150 mg/kg and the entire wound tissue was removed along the edge of the wound and total RNA was extracted. According to the transcriptome sequencing analysis steps, the Agilent 2200 Tape Station system was used for transcriptome sequencing of the samples at each time point in each group. The sequencing results were analyzed through multiple database comparisons to obtain different gene types and expression levels at different time points in different groups, as well as different pathway information.

#### 2.2.12 RT-PCR analysis

Col I and Col III Gene sequences were obtained from Gene Bank and input into Primer5.0 to design specific primers. Using the total RNA extracted from 1.2.11 as the template, reverse transcription kit was used to reverse transcribed into cDNA, and real-time quantitative fluorescence detection was carried out according to the reaction system in Table 3 and procedure in Table 4, to observe the dynamic change process of the defect area and surrounding defect area of the two kinds of collagen in the samples of each group at various time points.

**TABLE 3:** RT-PCR procedure.

Procedure	Cycles
95°C, 10 min	1
95°C: 10 s; 60°C: 1 min; 95°C: 15 s	40
60°C: 1 min	1
95°C: 15 s-0.05°C: 5 s	STOP

**TABLE 4:** Cytotoxicity evaluation grading (Wang et al., 2022).

Relative propagation rate (%)	Cytotoxicity grading	Outcome assessment
≥100	0	non-cytotoxic
75–99	1	non-cytotoxic
50–74	2	Mild cytotoxicity
25–49	3	Moderate cytotoxicity
1–24	4	Moderate cytotoxicity
0	5	Severe cytotoxicity

#### 2.2.13 Statistical analysis

The values were expressed as the mean ± standard deviation (SD). Statistically significant differences (*p* < 0.05) among the different groups were evaluated using Student’s t-test and one-way analysis of variance with Tukey’s *post hoc* multiple comparison test. All of the statistical analyses were performed using SPSS 19.0 software.

## 3 Results and discussion

### 3.1 State and structure of collagen sponge

**FIGURE 1:**
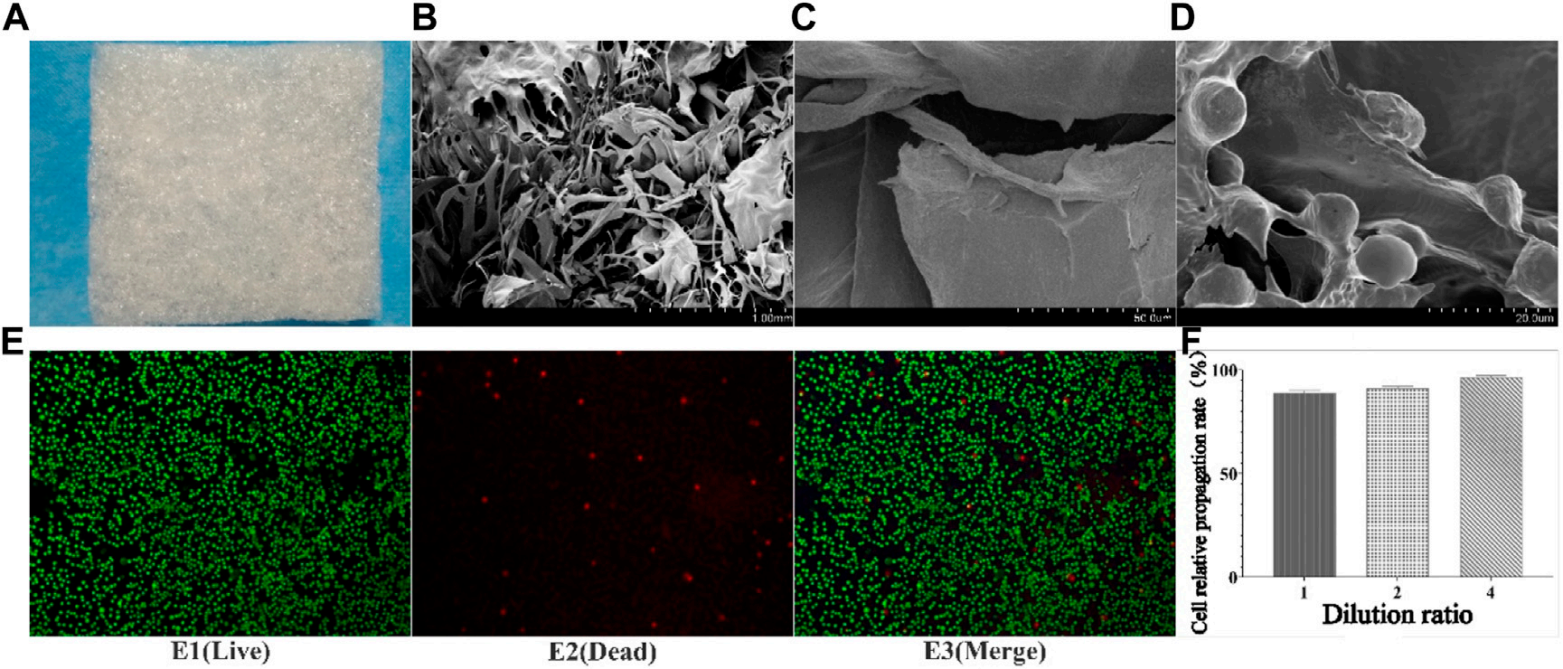
Results of collagen preparation, SEM, and co-culture with cells. **(A)** Collagen sponge; **(B)** SEM results of collagen sponge (Low multiples); **(C)** SEM results of collagen sponge (high multiples); **(D)** Crawl and growth of cells inside collagen sponge; E1-E3: Staining results of live/dead cells after 3 days of co-culture of sponge and cells; F: Relative proliferation rate of cells).

The collagen sponge prepared from tilapia concentrate was milky white porous sponge (Figure 1A), which has good elasticity and water absorption rate was about 1900% in the dry state (Wang et al., 2022). The results of scanning electron microscopy (Figure 1B/C) showed that the sponge was in the form of a porous honeycomb with a pore size between 20 μm and 120 μm, and the pore size was suitable for cells to enter and crawl.

### 3.2 Cell growth and adhesion state

Cytotoxicity is one of the important methods of inspection of medicinal materials are safe. Tested results found that different concentrations of fish collagen sponge leaching solution under the intervention of cell growth condition was good, cell proliferation rate above 90% (Figure 1F), reference cell toxicity grade evaluation criteria (Table 2), the cytotoxicity of collagen sponge for level 1, That was no cytotoxicity and had good cytocompatibility. To further verify the compatibility of cells with the material, live/dead cell staining and scanning electron microscopy were used to observe the adhesion and growth of cells on the surface and inside of the material (Figure 1E). The staining results showed that the collagen sponge with its excellent biocompatibility, high porosity, and high specific surface area, provided more cell binding sites, which facilitated the growth of cells into the material along the pore and scaffold structure, and continued to proliferate. The scanning electron microscope results further confirmed the crawling and growth of cells in the collagen sponge. It can be seen that a large number of cells adhere to the surface of the internal structure of the material (Figure 1D), and protruded pseudopodia to crawl and divided, indicated that cells can moved along the collagen surface and the pore structure, divided, grew and reproduced, proved that the material has good biocompatibility, good spatial structure and cell binding sites, and no cytotoxicity.

### 3.3 Model preparation and wound healing

**FIGURE 2:**
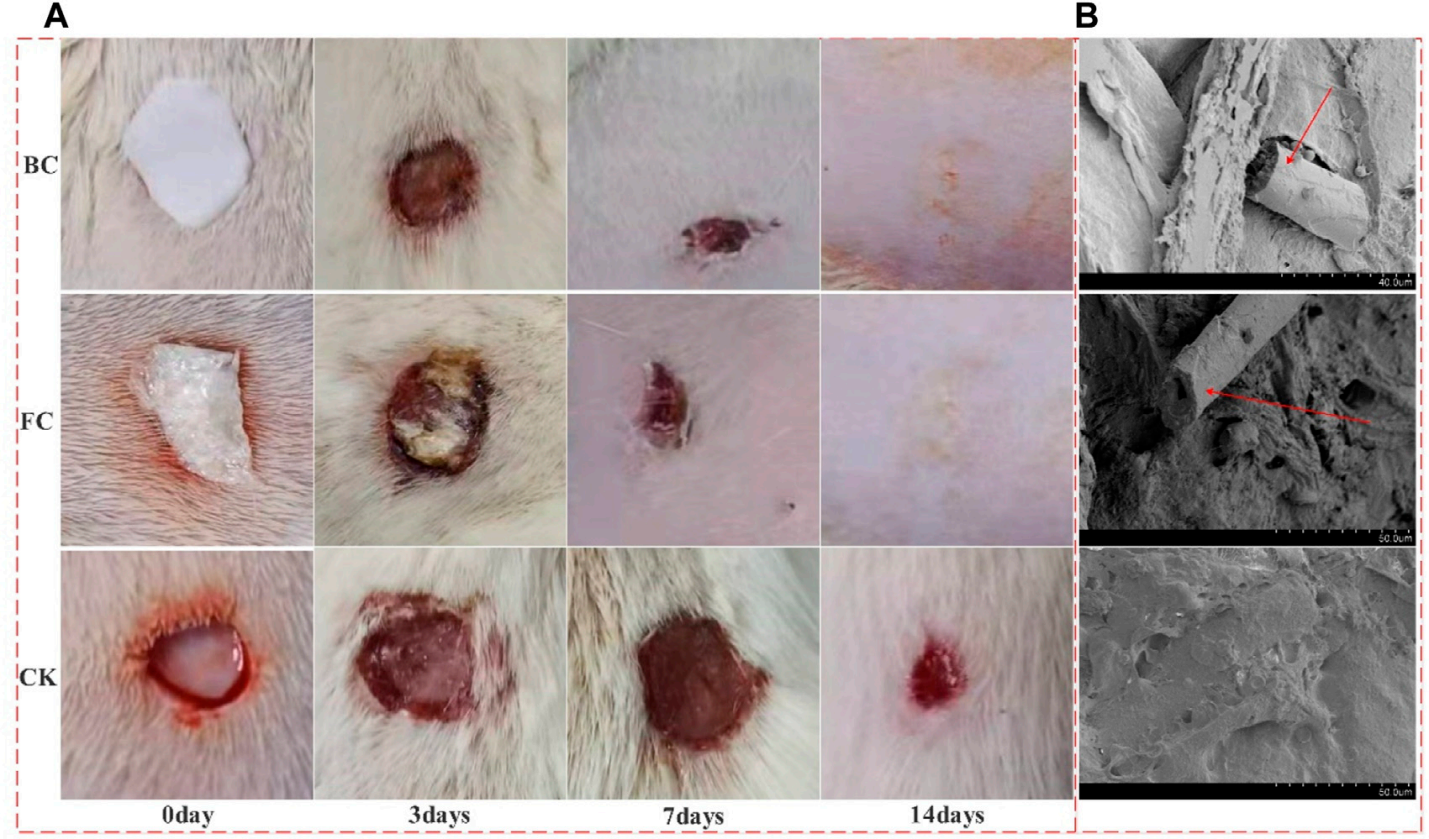
The different stages of wound healing and Vascular ingrowth. **(A)** Wound healing results; **(B)** Vascular ingrowth in the two collagen groups and self-healing group 24 h after surgery; BC: Bovine collagen sponge; FC: Fish collagen sponge; CK: self-healing group; The red arrow in the electron microscope image shows the neovascularization).

After the fish collagen sponge (FCS) was placed on the wound, it absorbed the oozing blood and tissue fluid in 30–60 s, and achieved good adhesion with the wound. After 2–3 min, a structure similar to blood scab was formed to achieve wound closure. After the bovine protein sponge (BCS) was placed on the wound, a similar phenomenon occurred, but the degree of binding to the wound was weaker than FCS (After collagen was combined with the wound, used tweezers to touch the collagen; Under the same strength, the combination degree of serious loosening or falling off is low). On the 3rd day, collagen was partially absorbed in both FCS and BCS groups. There was no significant difference in the area of wound reduction between the two groups, but remaining wound area of both were significantly smaller than control group. On the 7th day, the wound area of FCS and BCS groups was further reduced, and the collagen covering position was higher than the surrounding skin tissue, and a large amount of granulation tissue could be judged to grow. On the 10th day, the scabs of some individuals in the fish collagen group fell off and the wounds were completely healed. On the 12th day, some individuals in the bovine collagen group had completely healed. On the 14th day, the wounds of all individuals in the FCS and BCS groups healed completely, However, the control group has not yet seen recovered individuals. It can be preliminarily judged that the fish collagen sponge and the commercially available bovine collagen wound repair sponge have similar wound repair ability, and both collagens have good healing ability (Figure 2A).

### 3.4 Vascular ingrowth

In the early stage of wound repair, the growth of microvessels and good blood supply are the key to wound repair, and the key to whether materials can promote wound repair. The 24 h electron microscope results showed that the FCS and BCS groups had relatively complete spatial structures, visible pore structures, many red blood cells and other type cells were in the materials, and microvascular structures appeared in some areas. At 48 h, the FCS and BCS were occupied by many tissues or blood scabs, and the spatial structure could not be clearly identified. The control group did not find a lot of blood vessel ingrowth at 24 h. Thus, it was shown that both collagen sponges could promote the growth of microvessels and provide a good blood supply for wound repair (Figure 2B).

### 3.5 Histopathological examination results

**FIGURE 3:**
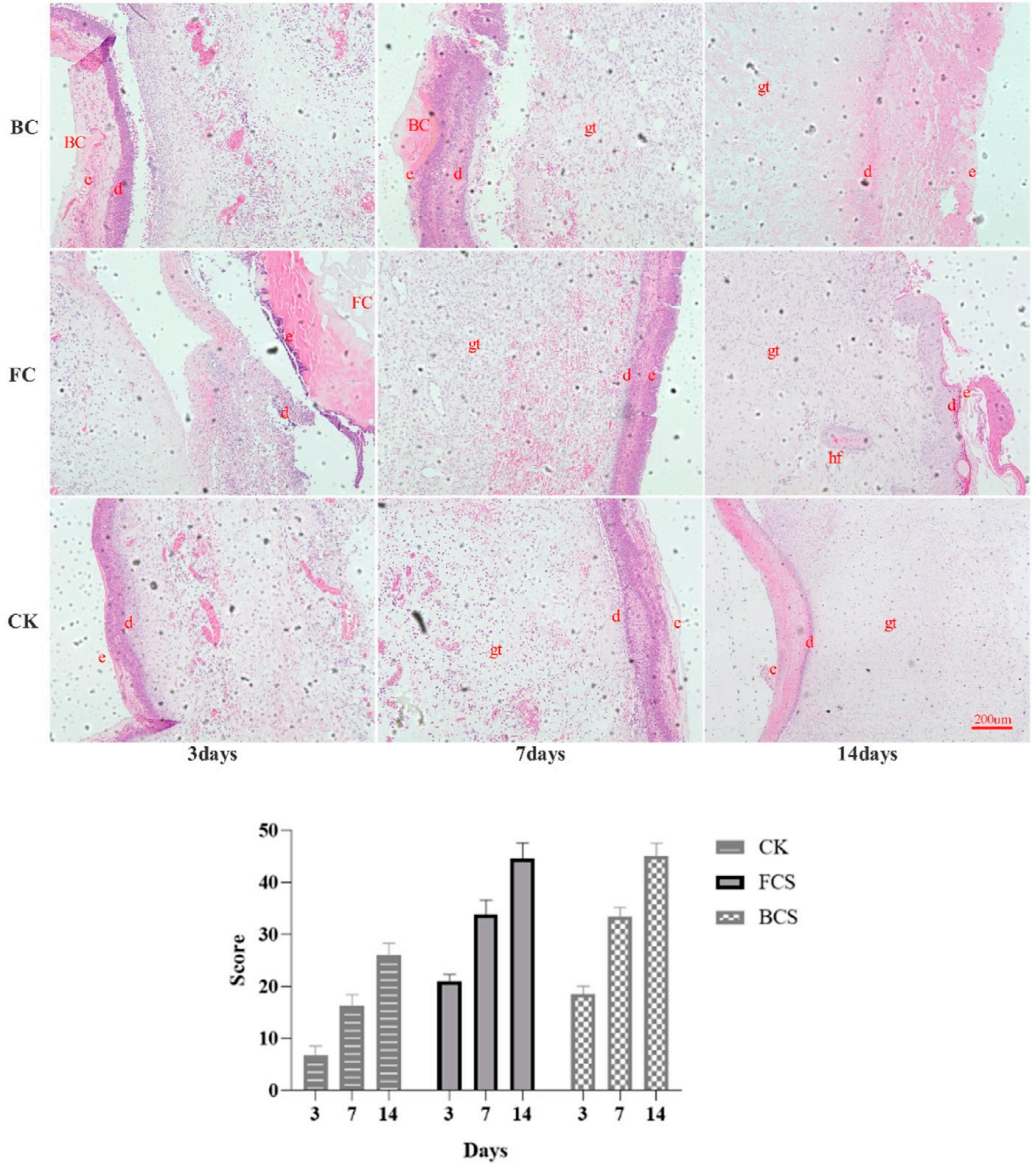
Analysis of HE stained would tissues (Magnification, ×100) and Scores. (The alphabetic letters e, d, gt and hf represent epidermis, dermis, granulation tissue, hair follicle, respectively. Scores in the histogram are the average scores of each time point calculated according to Table 4).

The results of HE staining (Figure 3) showed that on the third day, a small number of inflammatory cells were found in the wounds of FCS and BCS groups; blood vessels, collagen fibers, fibroblasts, etc. Appeared in some areas; the number of granulation tissue and fibroblasts was significantly higher than control group. In the control group, there were a large number of inflammatory cells in the wound, and no vascular tissue appeared. On the 7th day, some skin tissue structures were seen in the wound areas of FCS and BCS groups. In the middle, there was a large amount of granulation tissue and angiogenesis, and many fibroblasts were located at the wound surface, and a large amount of new collagen was deposited in the wound surface; In the control group, many inflammatory cells were still seen; the number of fibroblasts, granulation, blood vessels and other tissue growth were significantly weaker than those in the two collagen experimental groups. On the 14th day, complete epithelial tissue was formed in FCS and BCS groups, and a dense scar tissue was formed in the central area of the wound, and the overall structure of the tissue in the defect area was close to normal tissue. The self-healing group was significantly weaker than the collagen experimental groups in terms of scar tissue density and collagen fiber thickness. According to the HE staining scoring system (Table 5), the wound healing at each time point was quantified, the average score of the fish collagen group was higher than that of the bovine collagen group at all three time points, indicated that the healing situation of fish collagen group was better than the bovine collagen group; the average score between the two groups and the control group at each time point was greater than 3 points, the wound healing effect of the FCS group and BCS group was significantly better than self-healing group at each time point (Figure 3).

**TABLE 5:** Histological evaluation of wound tissue in all groups by H&E staining.

	0	1–3	4–6	7–9
Inflammatory cells	Abundant	Moderate	Scant	Rarely
Fibroblast content	None	Scant	Moderate	Abundant
Re-epithelialization	None	Partial	Thin	Complete
Collagen deposition	None	Scant	Moderate	Abundant
Revascularizations	None	Scant	Moderate	Abundant

**FIGURE 4:**
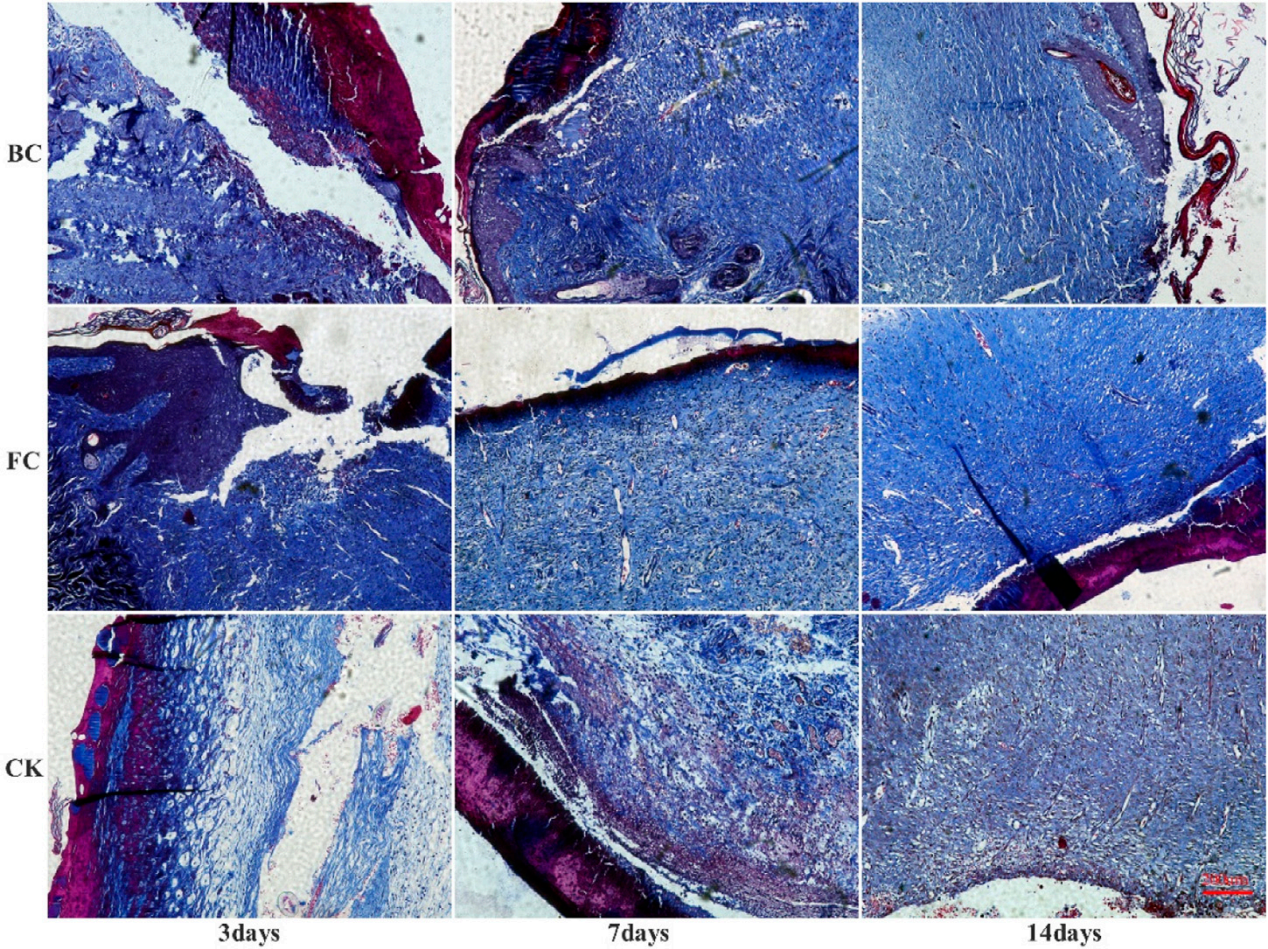
Analysis of Masson stained would tissues (Magnification, ×100).

Masson staining (Figure 4) showed that on the 3rd day, the three groups had formed reticular collagen fibers, FCS group had the highest fiber density, followed by the BCS group, and the self-healing group had the lowest density. On the 7th day, the granulation tissue grew in a good state in each group, the density of the network structure was further increased, and the collagen fibers thickened significantly. On the 14th day, FCS group had formed a dense structure with uniform thickness and almost no pores; BCS group was in the middle of the collagen fibers, occasionally larger pores were seen, but the collagen fibers had reached a certain degree of density. The fiber diameter and density of self-healing group were significantly smaller than those of the two collagen groups.

### 3.6 Expression of col I (type I collagen), VEGF and FGF

**FIGURE 5:**
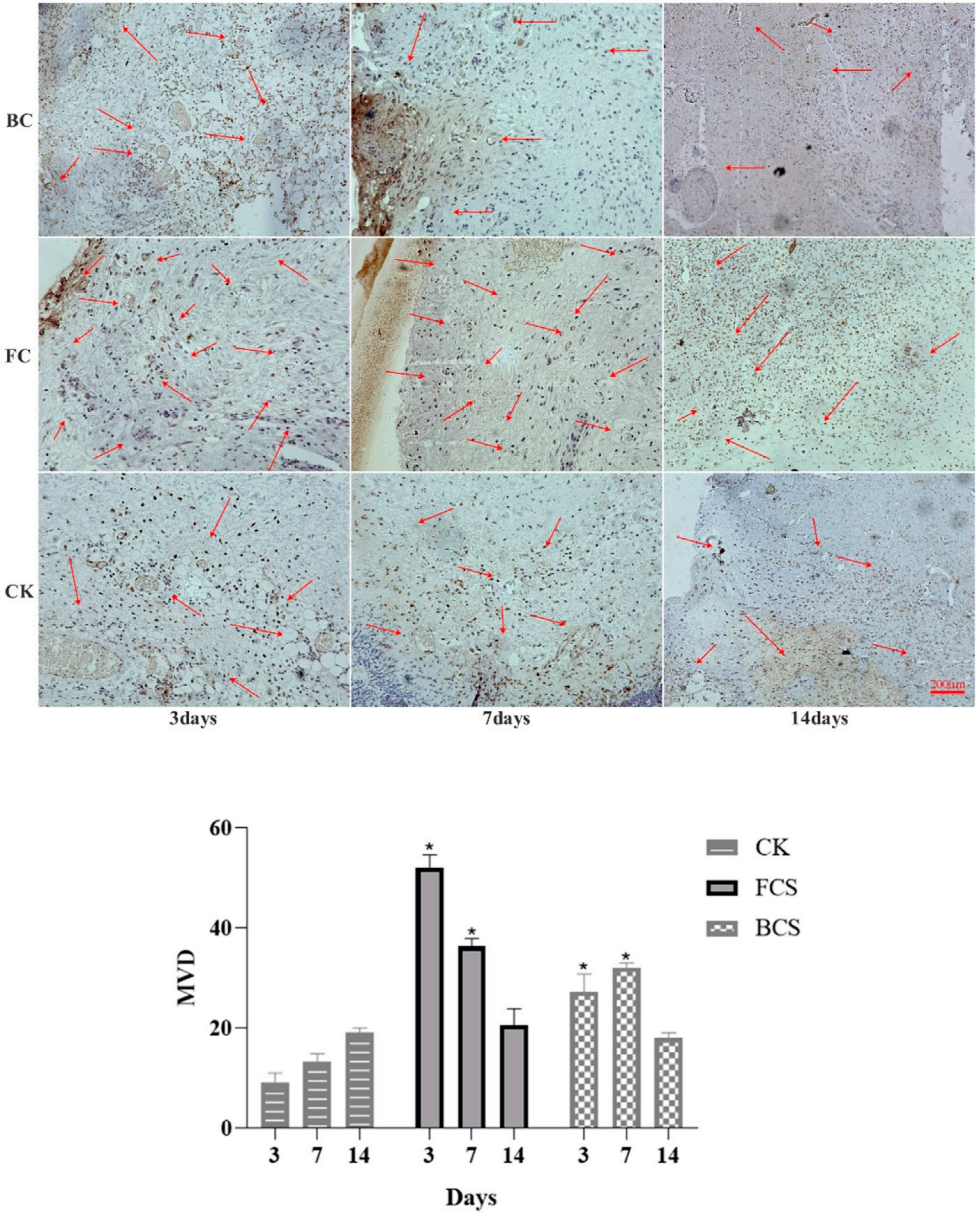
Immunohistochemical analysis of VEGF expression in wounded tissues (Magnification, ×100). The red arrow shows the new blood vessel. The histogram summarizes the microvessel density (MVD), which was determined by immunohistochemical staining for VEGF. (Each bar represents the mean ± SD. *p < 0.05: significantly different from the control group).

**FIGURE 6:**
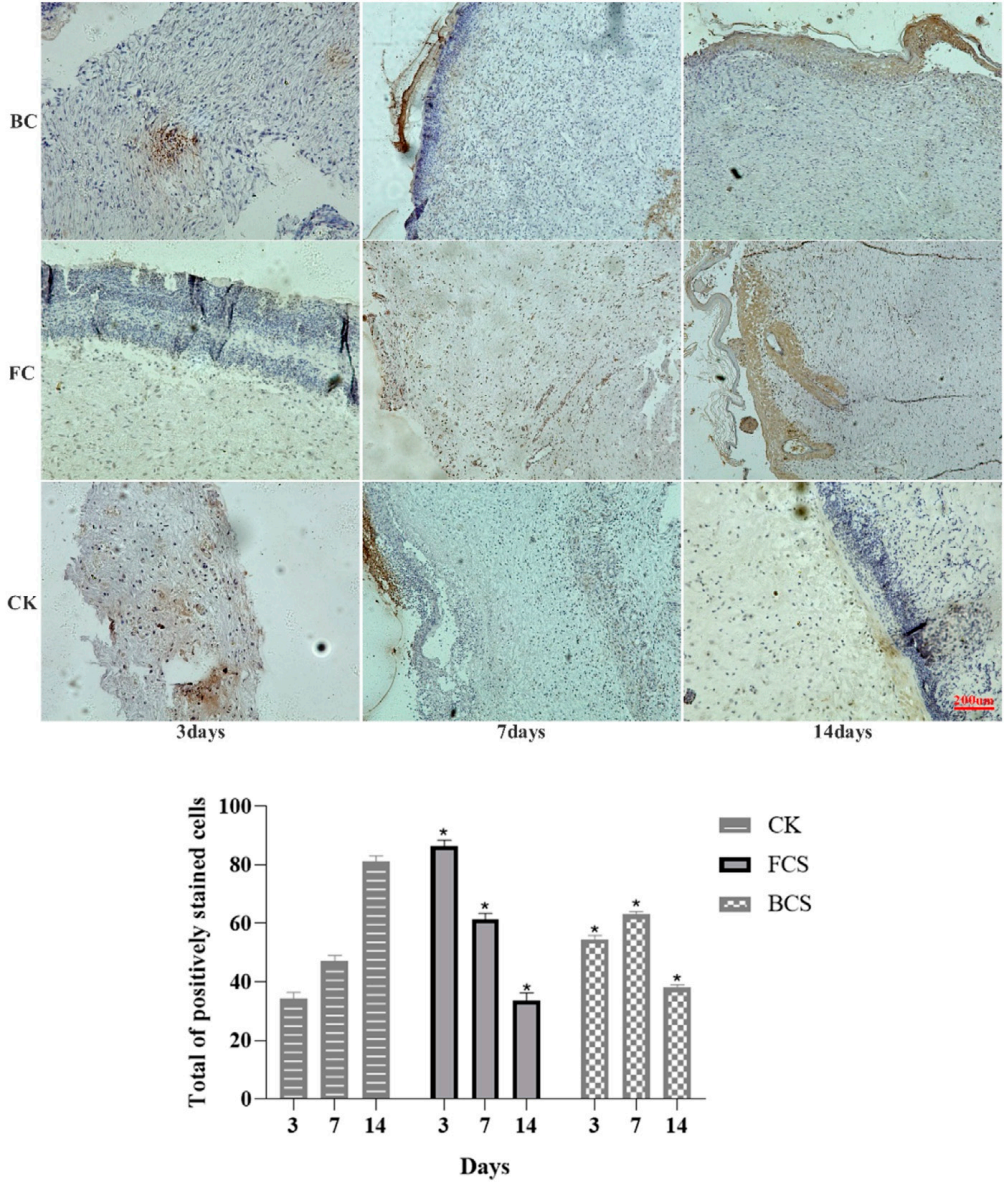
Immunohistochemical analysis of FGF expression in wounded tissues (Magnification, ×100). Note: FGF is mainly expressed in fibroblasts, which are colored during immunohistochemistry and distributed in the surface, middle and bottom layers. the histogram showed the total of positively stained cells of FGF in the dermal tissue per group. (Each bar represents the mean ± SD; * *p* < 0.05: significantly different from the control group).

**FIGURE 7:**
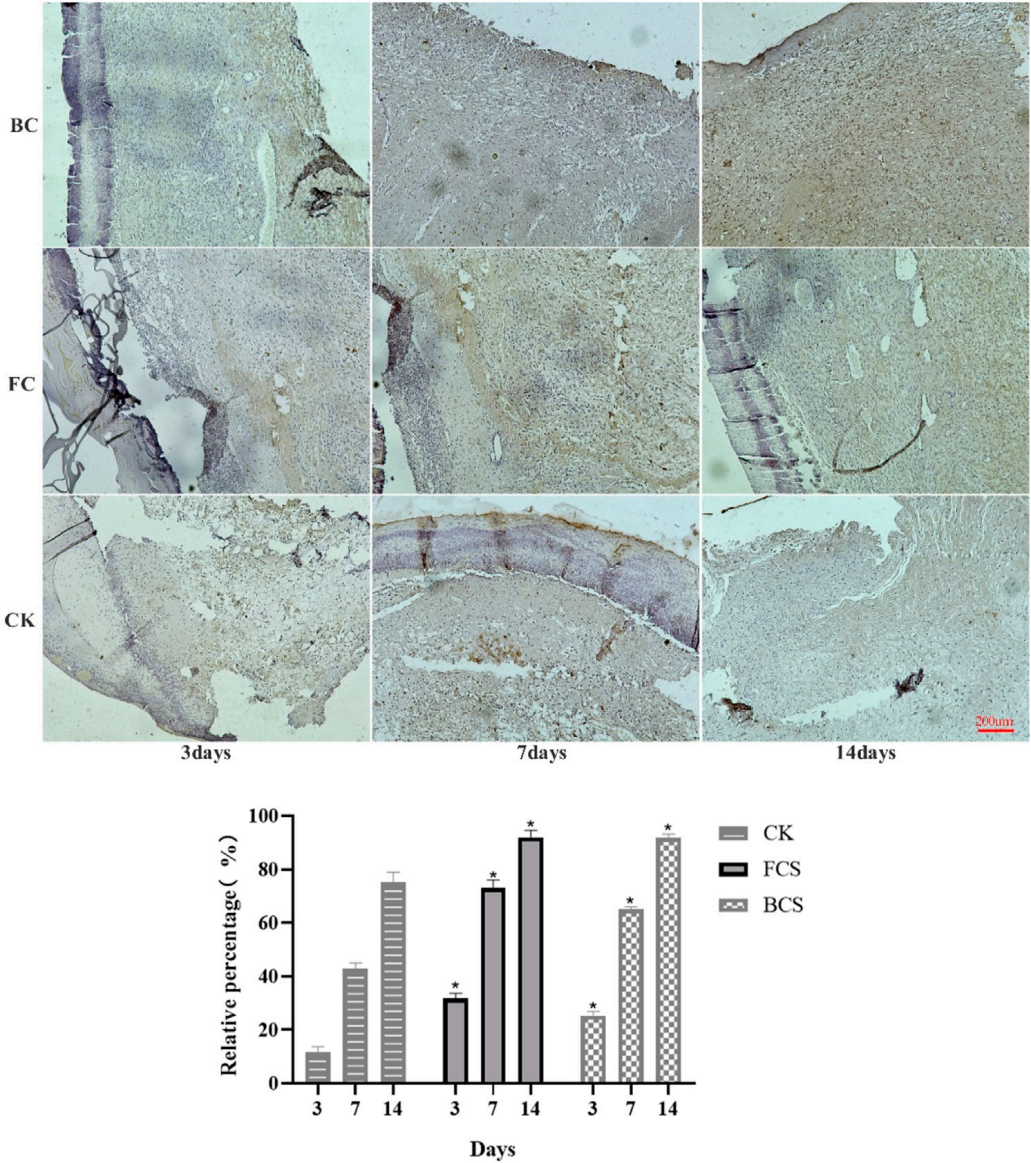
Immunohistochemical analysis of FGF expression in wounded tissues (Magnification, ×100) Note: FGF is mainly expressed in fibroblasts, which are colored during immunohistochemistry and distributed in the surface, middle and bottom layers. The histogram showed the total of positively stained cells of FGF in the dermal tissue per group. (Each bar represents the mean ± SD; *p < 0.05: significantly different from the control group).

VEGF (vascular endothelial growth factor) is a highly specific vascular endothelial cell growth factor, which has the functions of increasing vascular permeability, promoting vascular endothelial cell migration and proliferation, and inducing angiogenesis and proliferation *in vivo*. It is of great significance to the establishment of early blood supply (Gao et al., 2021; Shukla et al., 2021). FGF (fibroblast growth factor) plays an important role in wound healing and can effectively promote the migration, proliferation, and vascularization of fibroblasts (Zhao et al., 2020; Chakrabarti et al., 2021). Immunohistochemical analysis showed that with time, the expression of VEGF in the fish collagen group (FCS) showed a downward trend; the bovine collagen group showed a trend of first increasing and then decreasing; and the self-healing group showed an increasing trend throughout the observation period. On day 3, the relative number of microvessels in fish collagen group (about 52 on average) was higher than that in bovine collagen group (about 27 on average), and both groups were significantly higher than that in self-healing group (about 7 on average). On day 7, there was no significant difference between the two groups (30–40 microvessels), but it was still significantly higher than the self-healing group (about 10 microvessels). On day 14, there was no significant difference between the three groups (Figure 5). The expression and differences of FGF among groups and at each time point were the same as those of VEGF (Figure 6). During the experiment period, the number of positive stained cells in FCS group decreased from about 85 to about 35. The BCS went from 25 to 32, then down to 18; The self-healing group continued to rise from 8 to 19. Type I collagen is the main component of the two collagen sponges, and plays an important role in the formation of granulation tissue and scar tissue, as well as the migration and proliferation of cells in the process of wound repair. The results of immunohistochemistry showed that the content and density of total type I collagen in the two collagen groups showed an upward trend. On day 3, the content of type I collagen in FCS group was about 35%, and that in BCS group was about 28%, which was significantly higher than that in self-healing group (10%). On day 7, the FCS group and BCS group were 70% and 63% respectively, which were also significantly higher than the self-healing group (42%). On the 14th day, with the increase of fiber density and diameter, the wounds in the two experimental groups shrank significantly, and the wound shrinkage in the self-healing group was smaller than the two experimental groups, and the content of type I collagen per unit area in the two experimental groups (FCS group 90% and BCS group 89%) was still significantly higher than the self-healing group (78%) (Figure 7). This showed that on the 14th day, the wounds of the two experimental groups had basically healed and formed smaller scar tissue, while the self-healing group had not yet completely healed and scarred.

### 3.7 Metabolism of collagen in rat skin wounds and *in vivo*

The peptide chain of collagen contains chromophores such as C-O, -COOH, CONH, etc. The absorption peak is detected in the near-ultraviolet region (Nalinanon et al., 2011; Tamilmozhi et al., 2013), and FITC has an absorption peak at 490 nm. The free ε-amino group of the lysine residue of the protein molecule can undergo a nucleophilic reaction with FITC to form a thiourea linkage and covalently bind to form a conjugate. The ratio of A490 and A280 can be used to calculate the quality ratio (F/*p*-value) of FITC and collagen in the final conjugate, to judge the effect of marking. After multiple tests, it was found that the F/P was in the range of 0.451 ± 0.037, indicated that the labeling effect was good.

**FIGURE 8:**
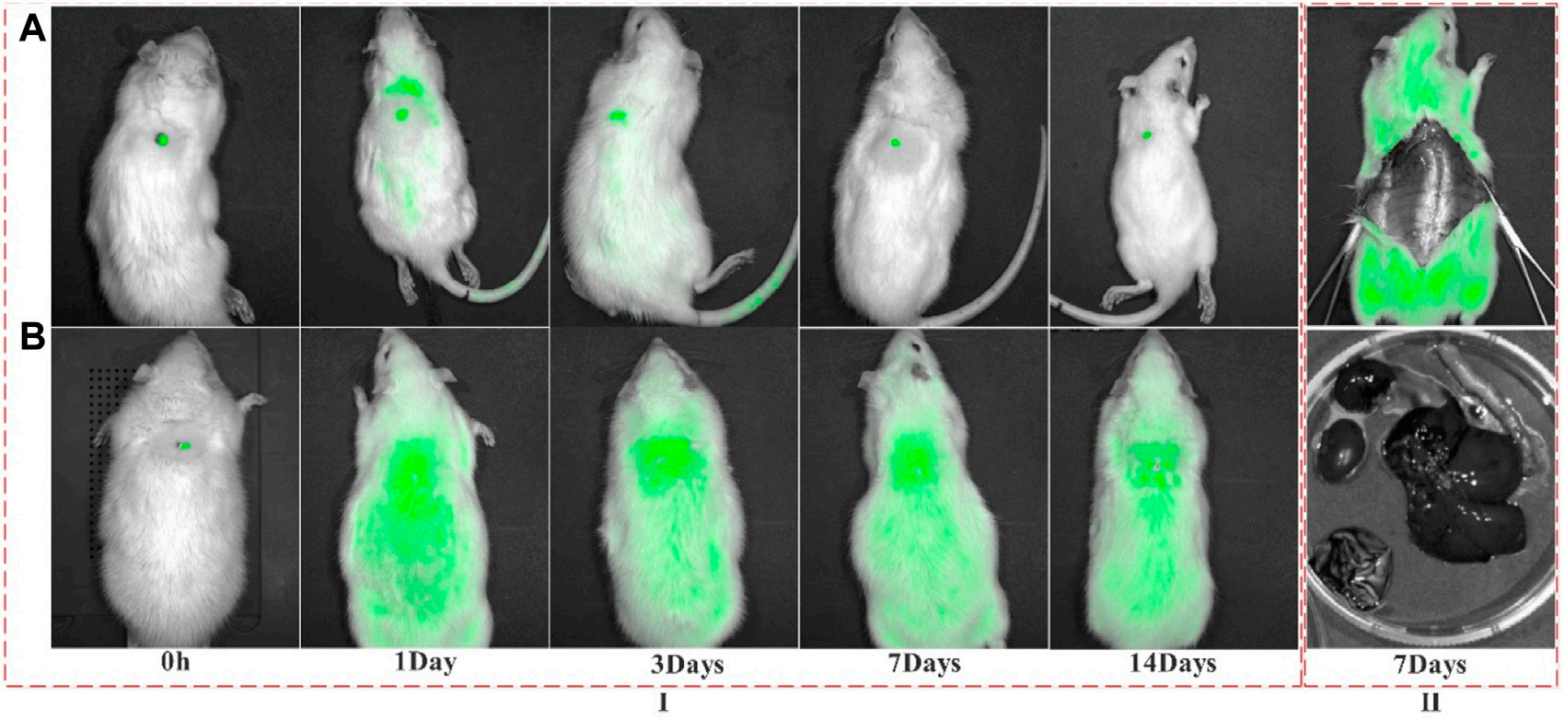
Fluorescence distribution of FITC and FITC-Col at different time points after implantation. **(A)** FITC-Col (FITC-labeled fish collagen); **(B)** FITC; I: Fluorescence distribution in the whole body of rats at different time points; II: Fluorescence distribution of different organs on 7th day). Note: The green fluorescence in this image shows FITC or FITC-labeled collagen.

It can be seen from the fluorescence images (Figure 8) that when the collagen and FITC was just implanted (0 h), the fluorescence in both the FITC-col group and the FITC group was at the wound location. On the 1st and 3rd day, in the FITC-col group, except for a small amount of fluorescence appeared in the trunk position outside the wound, high-intensity fluorescence was concentrated in the defect area, while in the FITC group, except for the high-intensity fluorescence at the wound position, there was a lot of fluorescence around the area and trunk. On the 7th day, most fluorescence of the FITC-col group was concentrated in the wound area, and there was almost no fluorescence in other parts. The entire back of the FITC group was centered on the wound surface, with a large number of fluorescent spots distributed from strong to weak and from inside to outside. On the 14th day, the fluorescence on the back of the rats in both groups was significantly weakened, but the fluorescence in the FITC-col group was mainly concentrated in the wound, while the fluorescence in the FITC group almost spread over the entire back trunk of the rats. In addition, in order to determine the whereabouts of fluorescent substances, on the seventh day, 3 rats were randomly se-lected from all the rats and sacrificed, their skin and metabolic organs were peeled off. It was observed that no fluorescence was found in all metabolic organs and the whole body, which preliminarily indicated that the free fluorescent markers were mainly excreted through skin metabolism, while FITC-col was utilized by the body at the wound site. It can be judged that the FITC fluorescent marker was gradually metabolized by the rat body as the wound heals, and does not participate in the wound healing process; while the FITC-labeled collagen was used in the healing process of the wound in the form of macromolecules or small molecules.

### 3.8 Distribution of fluorescence in wound tissue

**FIGURE 9:**
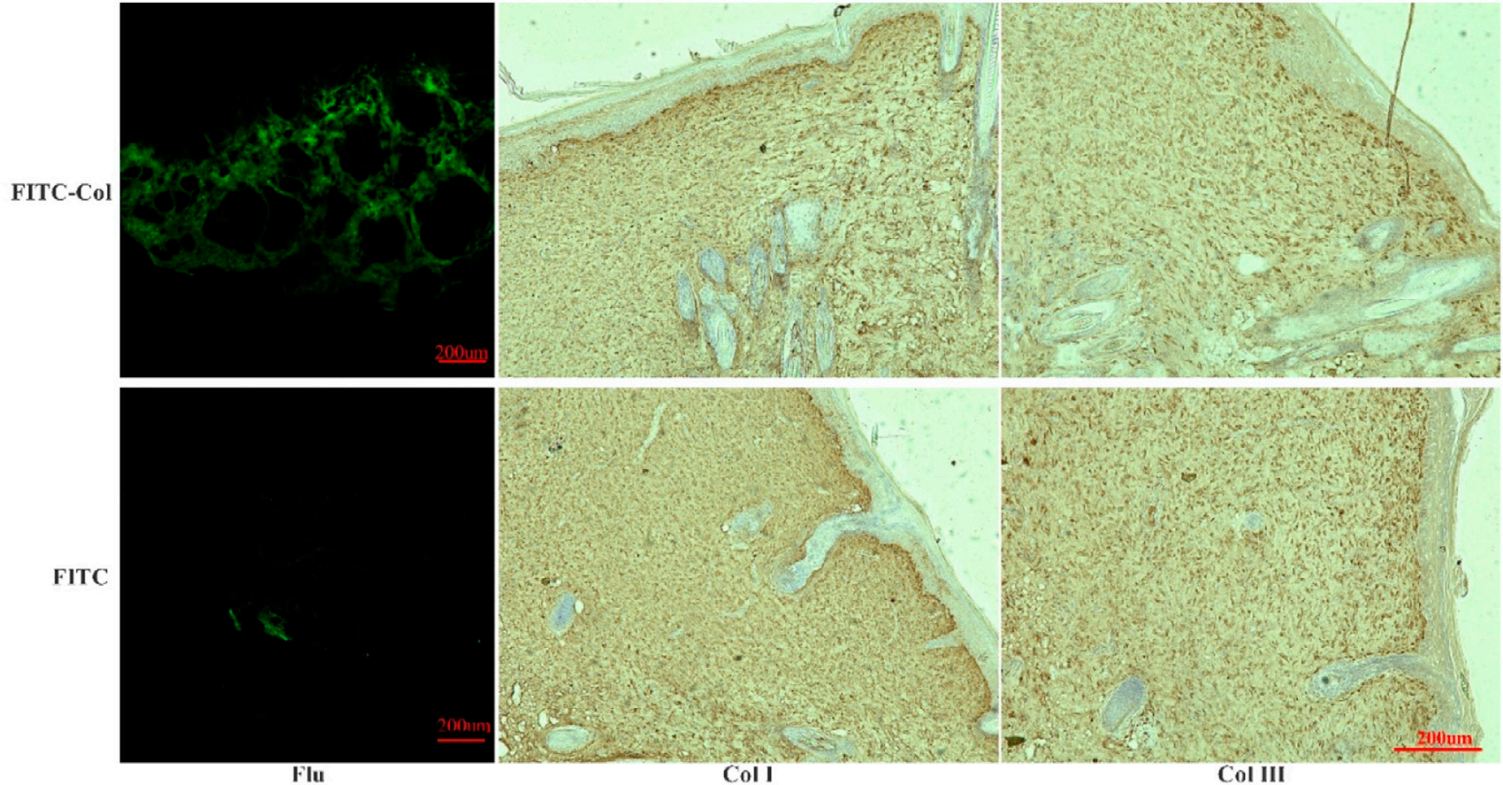
Fluorescence observation in frozen sections and immunohistochemical analysis of Col I and Col III. (FITC-Col: FITC-labeled fish collagen; FITC: FITC fluorescein; Flu: Fluorescence observation results of frozen sections; Col I: Immunohistochemical results of Col I; Col III: Immunohistochemical results of Col III).

Fluorescence observation of frozen tissue sections showed (Figure 9) that a large number of fluorescence was scattered at different structural positions in wound tissues of FITC-labeled fish collagen group. After immunohistochemical treatment of the sections, it was found that fluorescence mainly occurred in the collagen fiber structure formed by type I and III collagen. It could be preliminarily determined that some of the amino acids or peptides carrying FITC in the peptide chain of fish collagen were utilized by the rat body in some form to participate in wound reconstruction and become a part of collagen fibers in the newborn tissue. In addition, immunohistochemical results showed that scar tissue composed of type I and III collagen fibers had also been formed on the skin wounds in the FITC-luciferin group, but fluorescence could hardly be observed in the corresponding tissues, thus the interference of free FITC-luciferin on FITC-labeled fish collagen group could be excluded. It further indicated that the fluorescence in the wound site tissues of the FITC-labeled fish collagen group was labeled on free amino acid groups, rather than free FITC luciferin.

Laser confocal fluorescence observation was consistent with the living of fluorescence observations. After wound repair, part of fluorescence remained at the wound site, indicating that fish collagen may be decomposed into large fragments or small fragments of peptide chains or amino acids at the wound site, and then been used by the body at the wound site for tissue reconstruction.

### 3.9 Transcriptome analysis results

**FIGURE 10:**
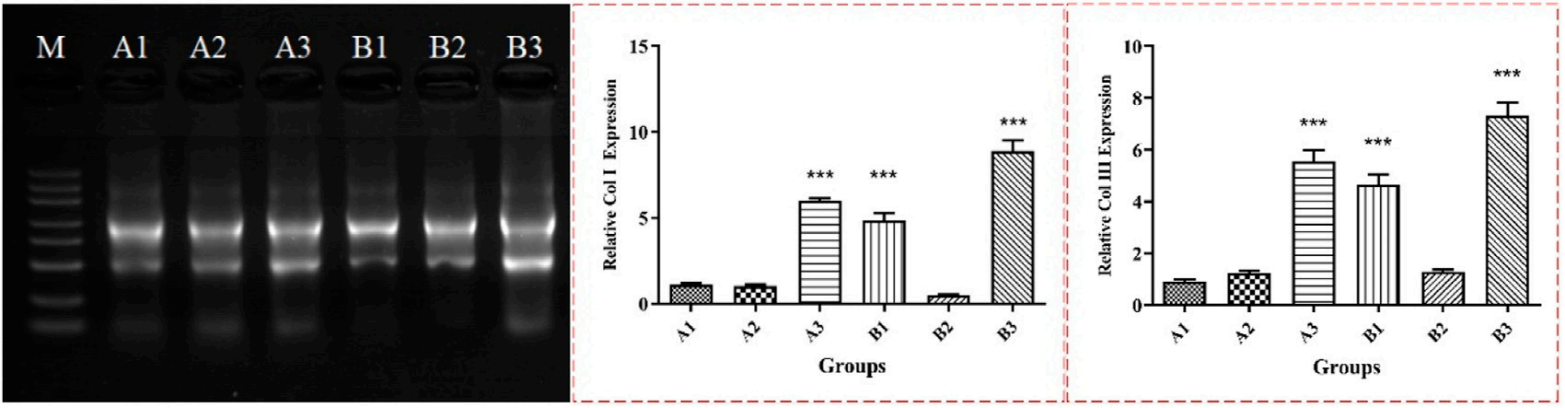
RNA electrophoresis results and relative expression levels of Col I and Col III. Note: *** represents significant difference in numerical comparison between the two groups at the same time point (*p* < 0.05). A1, A2 and A3 were corresponding to wound tissue samples of fish collagen group on day 1, 3 and 7. B1, B2 and B3 correspond to wound location tissue samples of the self-healing group on day 1, 3 and 7.

As can be seen from Figure 10 the electrophoresis patterns of total RNA extracted from most samples showed clear bands of 28S, 18S and 5S, and no other diffuse bands, indicated that the extracted RNA was complete and had good quality. Transcriptome sequencing results showed that the expression profiles of fish collagen group (group A) and self-healing group (group B) at different time points were independent of each other, with good repeatability within the group. According to the original read count value and FPKM value analysis of all samples, the five genes with the most significant expression differences were type I collagen, type III collagen, mitochondrial encoding cytochrome C oxidase, stearoyl-co A dehydrogenase, and cysteine-rich osteinin acidic secretory protein. Among them, type I and III collagen were the main structural proteins in granulation tissue, scar tissue and normal skin tissue during wound reconstruction. According to the analysis of FPKM values of the two collagen proteins (Table.6), during the postoperative observation period, the FPKM values of collagen type I in the fish collagen group showed a trend of first increase, then decrease and then increase, while the FPKM values in the self-healing group showed an upward trend. At the same time point, the FPKM values in the fish collagen group were significantly lower than those in the self-healing group (*p* < 0.05). It indicated that the expression level of type I collagen was significantly different between the two groups at the same time point. After surgery, the expression of type III collagen on the wound surface of the two groups showed different trends. On day 1, the FPKM value of the self-healing group was more than twice that of the fish collagen group, and the expression level of the two groups was significantly different. On day 3, the FPKM value of the fish collagen group increased slightly, while the FPKM value of the self-healing group decreased significantly, indicated that the expression level of the self-healing group decreased significantly at this time point, and the difference between day 3 and day 1 of fish collagen group was not significant, indicated that the expression level of the two groups at this time point was not significant. On day 7, the FPKM values of both groups were significantly increased, but the self-healing group was still significantly higher than the fish collagen group (*p* < 0.05), indicated that the expression level of type III collagen in the tissue samples of the defect area in the self-healing group was also significantly higher than that in the fish collagen group at this time point.

**TABLE 6:** Average FPKM values of type I and III collagen in wound tissues.

Days	FPKM			
	Fish collagen group		Self-healing group	
	Col I	Col III	Col I	Col III
1	1876.2663	1475.2341	3144.6392	3107.5829
3	1463.0591	1540.0778	4525.8021	1532.6934
7	6101.9033	5277.4549	11,527.8910	8977.0544

According to the transcriptome sequencing data, it can be preliminarily judged that the gene expression levels of type I and III collagen at the wound surface of rats changed greatly due to the implantation of exogenous collagen. The main manifestations were as follows: at different time points, the expression levels of type I and type III collagen in the wound tissues of rats implanted with fish collagen protein were significantly lower than those in the self-healing group. Presumably, Fish collagen after implanted in the rat skin wounds, involved the reconstruction of rat skin defect area in some form, mainly been used in type I and type III collagen, some peptides or amino acid was directly as a part of the body type I and III collagen, and feedback the signal to the body, caused the body to reduce the expression of related genes.

### 3.10 RT-PCR results

As can be seen from RT-PCR results (Figure 10), the change trend of the expression levels of the two collagen proteins in the wound site tissues of the fish collagen group and the self-healing group at different time points was the same as the change trend of the original read count value and FPKM value in the RNA transcriptome sequencing data, and the differences between the two groups at different time points were also the same. It was demonstrated again that the changes in the expression levels of type I and type III collagen were caused by the implantation of fish collagen at the wound site in rats, and the expression levels were much lower than those of the self-healing group. It further indicated that the presence of fish collagen at the wound site leads to changes in the signal transduction and pathway of wound repair, resulting in changes in the expression levels of the two collagen proteins.

## 4 Discussion

As the structural and functional component of dermal extracellular matrix, collagen is widely used in tissue engineering materials (Lim et al., 2019). These materials are regarded as natural biological repair material because of similar spatial structure to the human body and which can support cell differentiation, migration, crawling, and proliferation (Shoulders and Raines, 2009; Sorushanova et al., 2019), and play a crucial role in wound healing and bone defect repair (Hayman, 2008; Pal et al., 2016; Al-Maawi et al., 2018). The tilapia collagen sponge used in this study can change the porosity and spatial structure of the sponge by adjusting the final concentration of the concentrate. The obtained sponge has a uniform texture and stable spatial structure, which was convenient for cell adhesion, crawling and proliferation. Scanning electron microscopy and Live/dead cell staining results could fully confirm the above properties of collagen sponges. These properties are of great significance for the induction of fibroblast proliferation, collagen deposition and maturation, and re-epithelialization. In the early stage of acute full-thickness skin defect repair in rats, fish collagen sponge and bovine collagen sponge also showed excellent ability to induce blood vessel ingrowth, which can promote blood vessel ingrowth into the wound, restore blood supply and deliver nutrients in the defect area. In the later stage, it can be found that the two collagens are significantly better than the self-healing group in terms of wound repair speed and scar tissue size, indicated that the prepared collagen sponge can promote wound healing and reduce scar tissue formation (Kumar et al., 2014; Davidson et al., 2020). HE, Masson staining, and immunohistochemical results of VEGF, Col I, FGF showed that fish collagen sponge had obvious advantages in inducing blood vessel growth, fibroblast proliferation, collagen fiber synthesis, and re-epithelialization of the defect area. In the process of wound healing, fish collagen is first fused with new collagen fibers, then gradually degraded, and replaced by new tissue. It can be preliminarily confirmed that the collagen derived from fish has the same or even better wound repair ability than bovine collagen, and has the potential to be used as a wound repair dressing.

Many studies have shown that collagen can be degraded, absorbed, and utilized after being implanted into organisms, but there is no clear evidence of its ultimate destination (Copes et al., 2019; Shi et al., 2020; Zou et al., 2020). Therefore, the free carboxyl and amino groups of collagen peptide chain were combined with fluorescein to form conjugated compounds, which were implanted into the wound site of rats. The dynamic changes of fluorescence in rat skin and *in vivo* were observed by using animal fluorescence imaging system, so as to preliminarily determine the metabolic pathway of collagen. The results showed that the single fluorescein diffused into the surrounding skin tissue in a short time, and there was almost no fluorescence at the wound site after wound healing. However, fluorescein labeled collagen only diffused into the surrounding skin tissue at the early stage of wound healing, and most of the fluorescein remained at the original wound site after wound healing. Therefore, it can be preliminatively determined that in the process of wound healing, most collagen was degraded at the wound, and the degraded products participated in the process of wound reconstruction and remained in the new tissue, except for a small amount of free fluorescein and some small molecules carrying fluorescein that diffused into the surrounding skin tissue. By immunohistochemistry and laser confocal fluorescence observation *in situ* comparison, it was reconfirmed that fish collagen sponge was involved in the new type I and type III collagen in some form at the location of the wound along with the healing process of the wound. This was basically consistent with the speculation about the role of collagen in wound repair in many studies.

Transcriptome sequencing results and RT-PCR results can be preliminarily inferred that the body activates the reverse regulation mechanism, changes the regulation of part of the pathway, and reduces the expression of type I collagen and type III collagen-related genes because of the implantation of fish collagen protein. However, because fish collagen was utilized by the body to participate in the reconstruction of new collagen, the decrease of related gene expression level did not result in the reduction of total collagen deposition in the wound defect area, but the collagen deposition was larger than that in the self-healing group, which reflected from the side that fish collagen was involved in the process of wound repair and the construction of new collagen. Its metabolism and mechanism of action will be further studied and discussed in the future.

## 5 Conclusion

In this study, the collagen concentrate of tilapia skin was used to obtain fish collagen sponge by cryogenic freeze-drying method, which has good spatial structure and biocompatibility and can effectively promote the healing of acute wounds in rats. In the process of wound healing, collagen sponge can induce vascular ingrowth, cell crawling and proliferation, promote collagen deposition and maturation, and gradually be decomposed and utilized at the wound site. As a part of the new tissue, it participates in skin regeneration, and is a good wound healing material.

## Data Availability

The original contributions presented in the study are included in the article/supplementary material, further inquiries can be directed to the corresponding authors.
